# Functional Diversity of Mitochondrial Peptidyl-tRNA Hydrolase ICT1 in Human Cells

**DOI:** 10.3389/fmolb.2021.716885

**Published:** 2021-07-16

**Authors:** I.V. Chicherin, S.V. Dukhalin, R.A. Khannanov, M.V. Baleva, S.A. Levitskii, M.V. Patrushev, P.V. Sergiev, P. Kamenski

**Affiliations:** ^1^ Department of Molecular Biology, M.V.Lomonosov Moscow State University, Moscow, Russia; ^2^ National Research Center "Kurchatov Institute", NBICS Center, Moscow, Russia; ^3^ Belozersky Institute of Physico-Chemical Biology, Lomonosov Moscow State University, Moscow, Russia; ^4^ Center of Life Sciences, Skolkovo Institute of Science and Technology, Skolkovo, Russia; ^5^ Department of Chemistry, Lomonosov Moscow State University, Moscow, Russia; ^6^ Institute of Functional Genomics, Lomonosov Moscow State University, Moscow, Russia

**Keywords:** mitochondria, translation, termination, regulation, apoptosis, cell cycle, proliferation, ribosome, signaling

## Abstract

Mitochondria are energy producing organelles of the eukaryotic cell, involved in the synthesis of key metabolites, calcium homeostasis and apoptosis. Protein biosynthesis in these organelles is a relic of its endosymbiotic origin. While mitochondrial translational factors have homologues among prokaryotes, they possess a number of unique traits. Remarkably as many as four mammalian mitochondrial proteins possess a clear similarity with translation termination factors. The review focuses on the ICT1, which combines several functions. It is a non-canonical termination factor for protein biosynthesis, a rescue factor for stalled mitochondrial ribosomes, a structural protein and a regulator of proliferation, cell cycle, and apoptosis. Such a diversity of roles demonstrates the high functionality of mitochondrial translation associated proteins and their relationship with numerous processes occurring in a living cell.

## Introduction

Energy metabolism is one of the most important processes in living systems. In eukaryotic cells, it is mainly realized in the mitochondria, which are often called the “power stations” of the cell. Mitochondria are semi-autonomous two-membrane organelles whose evolutionary ancestors were alpha-proteobacteria. Their main role is the biosynthesis of ATP, which is produced by ATP synthase during the dissipation of the gradient of H^+^ ions generated in a complex chain of redox reactions on the inner membrane of organelles.

In addition to energy metabolism, mitochondria are involved in a number of other biosynthetic and signaling processes. The key one is apoptosis, a program that controls cell death. This process has two ways to activate (Ashkenazi et al., 2017). The first of them, external, is associated with the interaction of cellular receptors with “death ligands.”. The second pathway, internal, is closely associated with disturbances in the work of mitochondria. It is characterized by oxidative stress, permeabilization of the outer membrane and release of cytochrome c into the cytoplasm, which triggers a cascade of proteolytic reactions (Delbridge et al., 2016). The importance of studying apoptosis is determined by its central position in the network of carcinogenesis processes, which is a global threat to public health in the modern world. Violations of the “cellular suicide” program contributes to the transformation of a cell into a cancerous one, and mitochondria obviously play a significant role in this.

Human mitochondria have their own genome, a circular DNA molecule, which encodes 13 membrane components of the respiratory chain complexes, two ribosomal RNAs and 22 transfer RNAs (Anderson et al., 1981). Their biosynthesis requires a functional translation apparatus. The mitochondrial translation system is structured according to the classical scheme observed in prokaryotes. It consists of standard steps such as initiation, elongation termination and recycling, and includes a number of canonical actors such as mRNA, tRNA, ribosomes, and translation factors (Ott et al., 2016). However, at each stage, features that make the process of protein biosynthesis in mitochondria unique in nature were found (Kummer and Ban, 2021). These specific features arose as an adaptation of the system to the aggressive oxidative environment of mitochondria and functional specialization for the synthesis of a limited set of highly hydrophobic membrane proteins (Levitskii et al., 2020). For example, human mitochondrial mRNAs do not contain the 5′ leader sequences characteristic of prokaryotic and eukaryotic mRNAs; mitochondrial tRNAs differ from the canonical form of the clover leaf and have shorter domains; and mitochondrial ribosomes have a significantly higher protein content, smaller rRNA domains, and large-scale architectural rearrangements compared to ancestral ribosomes of prokaryotes (Greber and Ban, 2016).

The pronounced differences from the bacterial predecessor are observed in the work of mitochondrial translational factors. Only two translation initiation factors have been found in human mitochondria - MTIF2 and MTIF3. The IF1 homologue is absent, and its role is played by one of the domains of the MTIF2 (Kuzmenko et al., 2014). In addition, the deletion of MTIF3 does not stop the protein synthesis of organelles, which indicates a shift in the functional role of the protein relative to the bacterial homologue (Chicherin et al., 2020). The set and functions of termination factors have also altered significantly. Bacteria use two canonical class I translation termination factors - RF1, which recognizes the UAA and UAG codons, and RF2, which recognizes the UAA and UGA codons (Tuite and Stansfield, 1994). The genetic code of human mitochondria differs from the nuclear one by a reassignment of several codons. In particular, the universal stop codon UGA encodes tryptophan, and the two arginine codons AGA and AGG are unassigned and used as frameshifting sites leading to termination (Chrzanowska-Lightowlers et al., 2011). To date, four presumable termination factors have been described in human mitochondria based on homology and retention of conserved domains: mtRF1a, mtRF1, C12orf65, and ICT1 (Kummer and Ban, 2021). The first of them is the canonical homologue of bacterial RF1 in mitochondria: it is capable of recognizing termination codons (UAA and UAG) and hydrolyzing the bond between the peptide and tRNA, while the role of the others was suggested to terminate translation in various emergency cases. This review focuses on the factor ICT1 in human mitochondria. The accumulated body of evidence indicates that a protein can be a participant in a large number of biochemical processes, simultaneously performing as structural, catalytic, and regulatory component. Example of ICT1 illustrates the diversity of roles of mitoribosomal proteins and follows the current trend of interconnectedness of a large number of processes in a living cell.

## ICT1 is a Termination Factor and a Structural Component of Mammalian Mitochondrial Ribosome

Originally named as DS-1, ICT1 first appeared in the scientific literature in 1995 in a cancer study (Van Belzen et al., 1995). The authors discovered ICT1 mRNA encoding a protein with a molecular weight of 24 kDa, consisting of 206 amino acids with an isoelectric point of 10.9. It attracted the attention of researchers as one of the genes whose expression was significantly decreased during the differentiation of HT29-D4 colon carcinoma cells. In addition, the amount of its homologue in the embryos of mice was several times higher than in the cells of adults; therefore, it was proposed to consider the protein as a marker of undifferentiated cells (Van Belzen et al., 1998).

**FIGURE 1:**
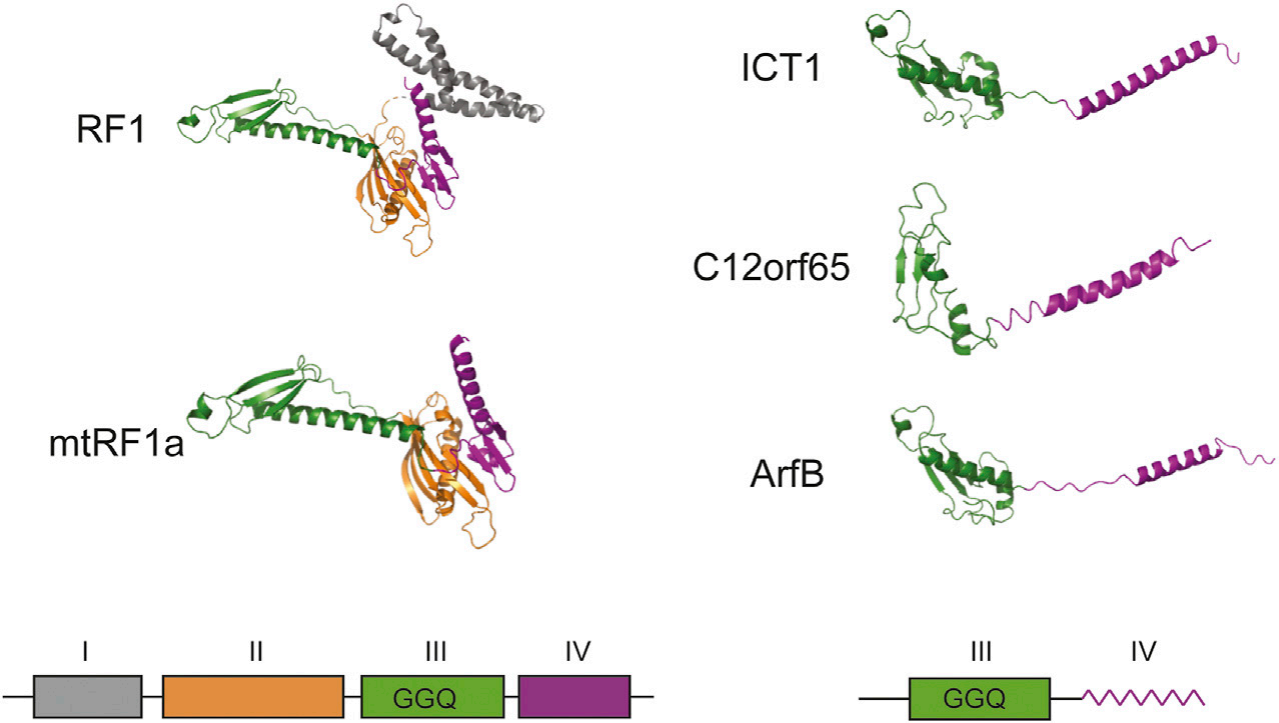
Schematic structural overview of homologous prokaryotic and mammalian mitochondrial translation termination and ribosome rescue factors. The corresponding schematic representation of the domain organization is given below. All structures were adapted from PDB: *Escherichia coli* RF1 (6DNC), *Homo sapiens* mtRF1a (7NQH), *H. sapiens* ICT1 (7NQL), *H. sapiens* C12orf65 (7A5H), *E. coli* ArfB (6YSS). No high-resolution structure is available for mammalian mtRF1, but it should be close to mtRF1a according to sequence similarity. I–domain of unknown function (grey), II–structural domain (orange), III–peptidyl-tRNA-hydrolase domain (green), IV–codon recognition domain (purple). In ICT1, C12orf65 and ArfB domain IV is schematically shown as a zigzag line to highlight its alpha-helical folding. The density of domain I is absent on the figure because it was not resolved in PDB: 7NQH. GGQ designates the three essential catalytic residues of peptidyl-tRNA-hydrolase domain. All structures are given as cartoon representations.

The next pages in the story of ICT1 were written by a research group at the University of Newcastle in the United Kingdom, which made a huge contribution to the study of translation termination in mitochondria. The research interests of the group were aimed at finding participants in the system of protein biosynthesis in mitochondria, as the data was limited. They used the methods of immunoprecipitation and mass spectrometry and analyzed the interactome of the mitoribosome recycling factor mtRRF (Rorbach et al., 2008). A large set of mitochondrial ribosomal proteins, as well as the ICT1, were found in the preparations. It turned out that ICT1 is imported into human mitochondria and interacts with the large subunit of the mitochondrial ribosome (Richter et al., 2010). Analysis of its amino acid sequence allows attributing it to a ribosome-dependent translation termination factors. Members of this family have several domains, among which the peptidyl-tRNA hydrolase and codon recognition domains are distinguished (Figure 1). While the first of them preserved as part of ICT1, the second one was completely lost. A similar picture is observed for another member of the family—C12orf65 (Kogure et al., 2012). As a result, the protein exhibits peptidyl-tRNA hydrolase activity regardless of the nucleotide sequence or even the presence of a codon at the A site. The activity is provided by three conserved residues GGQ of the hydrolase domain and is almost completely lost when they are mutated (Richter et al., 2010).

The functional role of termination factors in human mitochondria, including ICT1, has long been debated. The search for putative candidate proteins for this role was carried out bioinformatically based on sequence comparison and preservation of structural motifs. Historically, the mtRF1 factor was first discovered, but so far no one has been able to demonstrate its activity *in vitro* (Zhang and Spremulli, 1998). Later, the mtRF1a protein was recognized as a canonical termination factor, since it contained all the necessary structural elements and possessed peptidyl-tRNA hydrolase activity at stop codons UAA and UAG (Soleimanpour-Lichaei et al., 2007).

Deletion of the ICT1 gene is lethal, and a decrease in its expression by RNA interference leads to disruptions in the work of mitochondria (Richter et al., 2010). However, the exact role of ICT1 is unknown, and the researchers advanced several possible explanations. According to one of them, the nonspecific peptidyl-tRNA hydrolase activity of ICT1 could be helpful during the dissociation of ribosomal complexes stalled on truncated mRNAs lacking a stop codon (Richter et al., 2010). Thus, the protein could return ribosomal particles to the translational cycle. The second version about the possible role of ICT1 was born from the unusual genetic code of human mitochondria. The fact is that two human mitochondrial genes, COI and ND6, end with codons AGA and AGG, respectively, but neither tRNAs that decode them, nor enzymes capable of specifically terminating protein biosynthesis on these codons have been found, so both codons remain unassigned. Several mechanisms have been proposed to resolve this conundrum. One of them suggests that an additional U residue is inserted into the sequence by means of mRNA uridyl editing, creating the canonical UAG codon (Nozaki et al., 2008), but this hypothesis still has no experimental support. According to the second proposal, the mitoribosome pauses at the AGA and AGG codons due to the absence of complementary decoding tRNA. During the pause, the ribosomal complex shifts -1 nucleotide down in the opposite direction, setting the UAG codon at the A site (Temperley et al., 2010). According to the third suggestion, the translation of COI and ND6 mRNA is terminated by ICT1. It turned out that the protein is highly homologous to the factor ArfB (formerly YaeJ) of prokaryotes (Figure 1), which implements one of the ways of rescuing ribosomes stalled on mRNA (Lind et al., 2013). ICT1 has a similar structure and substrate specificity, in addition, both factors are able to complement each other’s deletions in bacterial and human cells (Feaga et al., 2016). However, ArfB terminate translation at truncated mRNAs lacking mRNA in the A-site and ICT1 is likely to perform the same role as suggested by the interchangeability of these factors. At the same time, AGA and AGG codons at the end of the COI and ND6 mRNA coding sequences are located at a distance of 72 and 500 nucleotides from the 3′-end of the corresponding mRNAs making an involvement of a termination mechanism similar to that of ArfB unlikely.

The physical interaction of ICT1 with mitoribosomes has been reported in several publications (Richter et al., 2010; Koc et al., 2013), however, the specific binding site has not been established. After the elaboration of high-resolution structures of human mitoribosomes, produced *via* cryo-electron microscopy (Brown et al., 2014; Amunts et al., 2015; Brown et al., 2017), ICT1 was found in the corpus of a large subunit. This came as a surprise, because *a priori* the protein was suggested to associate with it only temporarily, like most translation factors, but not as a structural component. In addition, it became obvious that its peptidyl-tRNA hydrolase domain obviously could not reach the peptidyl-transferase center making the speculations about its role in translation termination confusing. Subsequently, it was found that only free, but not ribosome-bound protein has enzymatic activity (Akabane et al., 2014). At the same time, no pool of unbound ICT1 has been ever found, so the mechanism of ICT1 functioning remains mysterious. Researchers have advanced several proposals. According to one of them, ICT1 is released from mitoribosomes stuck on mRNA for various reasons, for example, due to disturbances in the incorporation of growing peptides into the membrane. According to another version, the expression of ICT1 can sharply increase in response to a stress signal emanating from stopped ribosomes, creating a pool of free protein with termination activity (Akabane et al., 2014). It is possible that ICT1 inserted into the ribosome acts on the neighboring ribosome under certain conditions. This division into two functional groups, structural and enzymatically active, is extremely unusual and is not found in other translational factors.

## ICT1 is a Regulator of Proliferation, Cell Cycle and Apoptosis Involved in Carcinogenesis

As we mentioned earlier, ICT1 was first discovered in the study of cancer markers and further its role in oncogenesis was actively investigated. Overexpression of ICT1 has been reported in many types of malignancies such as glioblastoma (Xie et al., 2015), diffuse large B cell lymphoma (Xie et al., 2017), colorectal cancer (Lao et al., 2016) prostate cancer (Wang et al., 2015), breast cancer (Chen et al., 2017; Peng et al., 2018), hepatocellular carcinoma (Chang et al., 2017), leukemia (Li et al., 2018), lung cancer (Huang et al., 2014; Wang et al., 2017a) and osteosarcoma (Pan et al., 2021). These data, obtained by various methods, such as RT-qPCR, Western blotting, and immunohistochemistry, make it possible to classify ICT1 as an oncogene and use it as a marker for medical diagnostics. Interestingly, a number of studies have demonstrated a negative correlation between the level of ICT1 expression in tumors and patient survival, as well as its relationship with the clinical manifestations of the disease (Lao et al., 2016; Chang et al., 2017; Xie et al., 2017; Pan et al., 2021). According to clinical statistics, the protein content was higher in large, late and metastatic tumors (Chang et al., 2017; Xie et al., 2017; Pan et al., 2021). Thus, a high content of ICT1 can indicate an aggressive development of the disease, an unfavorable prognosis, and high risks to the life and health of the patient.

A homozygous ICT1 deletion is lethal to cells and no corresponding model exists. However, there are many reports about knockdown of ICT1 using an RNA interference system. Almost all of them indicate that a decrease in ICT1 expression leads to inhibition of proliferation of various types of cancer cells, as can be judged by counts of the number of cells in culture, building growth curves and comparison of cell colony sizes (Wang et al., 2015; Xie et al., 2015; Lao et al., 2016; Wang et al., 2017a; Wang et al., 2017b; Chang et al., 2017; Chen et al., 2017; Xie et al., 2017; Li et al., 2018). This is likely due to a downregulation of proliferative proteins such as PCNA and Survivin (Xie et al., 2017). In addition, depletion of ICT1 suppresses the migration of colorectal cancer cells (Lao et al., 2016) and gastric cancer cells (Tao et al., 2017), while its overexpression respectively leads to an increase in their mobility. Interestingly, the growth and division of non-cancerous cells, for example, osteoblasts, practically do not change upon ICT1 knockdown (Pan et al., 2021); therefore, the described phenomena are specific to tumor cells.

All reports devoted to the knockdown of ICT1 in cancer cells indicated that this procedure causes arrest of the cell cycle. Most of them claim that it occurs in the G_2_/M transition phase (Wang et al., 2015; Lao et al., 2016; Wang et al., 2017b; Chang et al., 2017; Chen et al., 2017). However, the data are not in complete accordance. Thus, in a study on the role of ICT1 in lung cancer, two cell cultures behaved differently in response to a knockdown. In culture 95D, arrest occurred in the G_2_/M phase, and in culture A549—mainly in the G_0_/G_1_ phase (Wang et al., 2017a). In the large diffuse B-cell lymphoma cell line RCK-8, ICT1 knockdown caused a cell cycle block in the G_0_/G_1_ phase (Xie et al., 2017). In human leukemia cells U937, no accumulation of cells arrested in the G_2_/M phase was found; however, an increase in S and sub-G_1_ populations was reliably observed (Li et al., 2018). Arrest in the sub-G_1_ phase was registered under similar conditions in glioblastoma cells (Xie et al., 2015). The discrepancies can be explained by the high heterogeneity of cancer cells, although, the conclusion about the effect of ICT1 on the regulation of the cell cycle has extensive experimental support. Apparently, the molecular mechanism underlying the control of the cell cycle by ICT1 is based on changes in the amounts of cyclins, cyclin-dependent kinases, and their inhibitor p21. According to experimental data, ICT1 knockdown causes an increase in the amount of p21 and a decrease in the amount of cyclin D1 in lung cancer (Wang et al., 2017a), a decrease in the amount of CDK1, CDK2 and cyclin B1 in breast cancer (Chen et al., 2017), a decrease in the amount of cyclins A2 and B1 in gastric cancer (Wang et al., 2017b), a decrease in the amount of CDK1 and cyclin B1 in hepatocellular carcinoma (Chang et al., 2017), a decrease in the amount of cyclin A2 and an increase in p21 in leukemia (Li et al., 2018). Overexpression of the protein accordingly leads to opposite effects (Chang et al., 2017).

**FIGURE 2:**
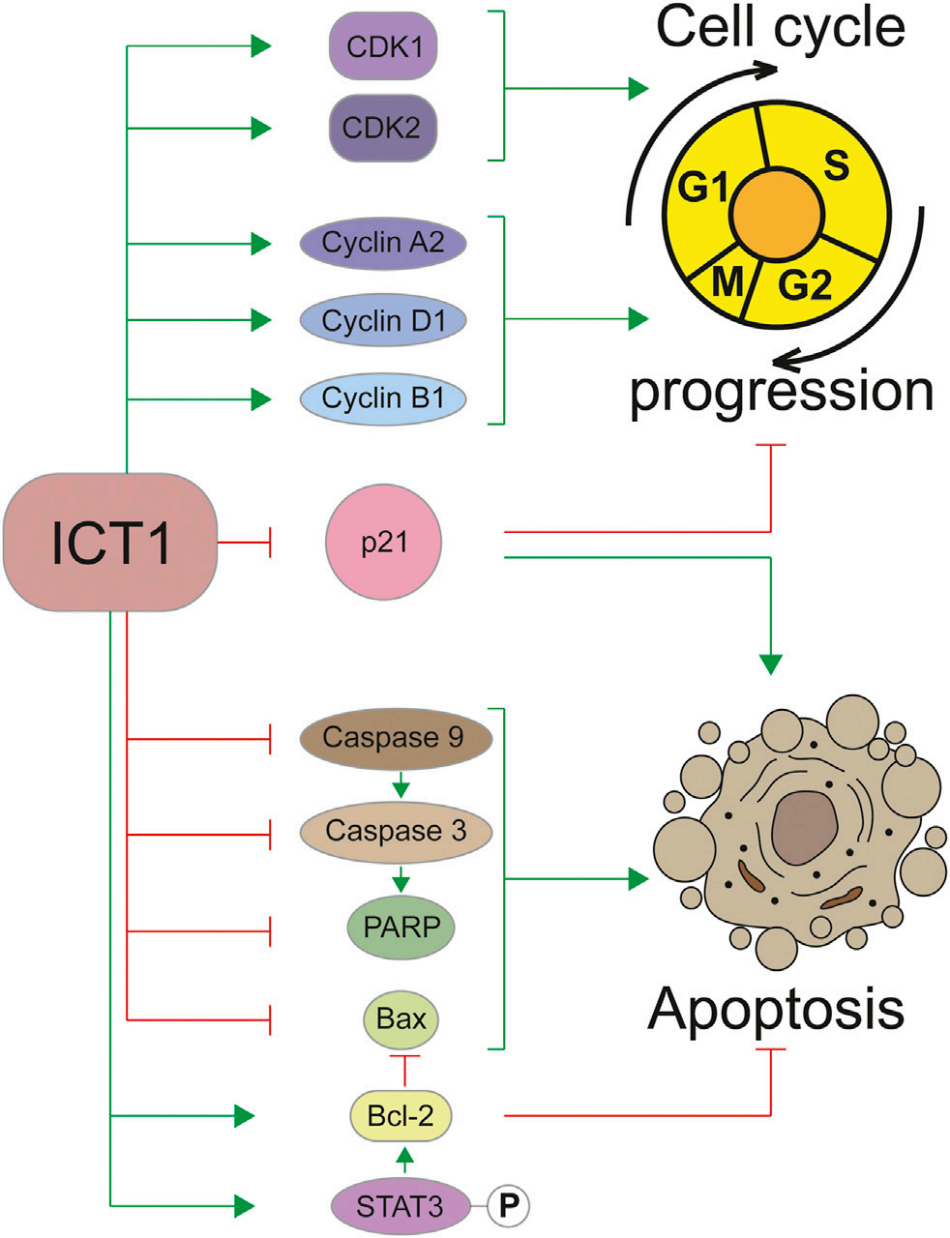
Regulation of the cell cycle and apoptosis by the ICT1 protein. Green arrowheads mean a promotion, red flatheads mean an inhibition. P in a circle represents a phosphate group.

Regulation of the cell cycle is closely related to another important biological process—apoptosis. Violation of this program largely determines the transformation of a cell into a cancerous one. The role of ICT1 in the regulation of apoptosis has been shown in a large number of studies carried out on various types of cancer cells, which allows considering it as a universal mechanism (Wang et al., 2015; Lao et al., 2016; Wang et al., 2017a; Wang et al., 2017b; Chang et al., 2017; Chen et al., 2017; Xie et al., 2017; Li et al., 2018; Pan et al., 2021). The high ICT1 content in cancer cells inhibits apoptosis, and its reduction through knockdown activates the “cellular suicide program.” Apparently, the regulation of apoptosis occurs due to the influence of ICT1 on the signal transmission pathways. A number of studies have demonstrated the interconnection of ICT1 expression with key genes regulating apoptosis, such as caspase 3, caspase 9, poly-ADP-ribose polymerase (PARP), Bax, and Bcl-2. Thus, a decrease in the expression ICT1 leads to proteolytic cleavage of the precursors and the accumulation of catalytically active forms of caspase 3 (Wang et al., 2015; Lao et al., 2016; Wang et al., 2017b; Chen et al., 2017; Xie et al., 2017; Li et al., 2018; Pan et al., 2021) and caspase 9 (Pan et al., 2021), as well as PARP (Wang et al., 2015; Lao et al., 2016; Xie et al., 2017) and Bax (Wang et al., 2015; Lao et al., 2016; Chang et al., 2017; Xie et al., 2017) cleavage. At the same time, the expression of the key inhibitor of apoptosis Bcl-2 is significantly decreased upon ICT1 knockdown (Wang et al., 2015; Chang et al., 2017; Chen et al., 2017; Xie et al., 2017; Pan et al., 2021). In addition to these data, the relationship between ICT1 expression and the phosphorylation status of key signaling molecules such as AMPK, Bad, PRAS40, JNK/SAPK was shown (Lao et al., 2016; Chen et al., 2017). Phosphorylation of STAT3, caused by ICT1 knockdown, is probably one of the mechanisms regulating Bcl-2, which determines the apoptotic status in cancer cells (Pan et al., 2021). It should be noted that ICT1 depletion does not suppress the migration of normal cells and does not induce apoptosis in them (Lao et al., 2016); therefore, the described processes are specific for carcinogenesis. The involvement of the ICT1 in the regulation of the cell cycle and apoptosis is shown schematically in Figure 2.

Micro-RNAs are generally recognized participants in the regulation of gene expression. Their role in human oncogenesis is also firmly established (Peng and Croce, 2016). Interestingly, the amounts of ICT1 correlate negatively with miR-1301-3p in breast cancer cells (Peng et al., 2018), miR-134 in hepatocarcinoma cells (Chang et al., 2017), and miR-205 in stomach cancer (Tao et al., 2017). In all cases, the microRNA content is less in cancer cells with high ICT1 expression. If the amount of micro-RNA is artificially increased, then the amount of ICT1 decreases, suggesting an inverse correlation. Simultaneously with a decrease in ICT1, overexpression of miR-205 and miR-1301-3p leads to phenotypic manifestations similar to those observed with depletion of ICT1, for example, suppression of proliferation and migration, arrest of the cell cycle and induction of apoptosis (Tao et al., 2017; Peng et al., 2018). These consequences can be prevented by artificially increasing the expression of ICT1, which indicates antagonistic interactions of the factor with microRNAs.

## Discussion

One of the key trends in modern molecular biology is the connection of multiple processes taking place in a living cell. This interplay is vividly illustrated by the example of the ICT1 protein, the main hero of this review. ICT1 is a mitochondrial protein and a structural component of the large subunit of the human mitoribosome. At the same time, it turned out to be a non-canonical translation termination factor in mitochondria, hydrolyzing peptidyl-tRNA regardless of the codon set in the A-site. Its biological role has not been definitively figured out. The factor could be used for rescuing stalled ribosomal complexes or completion of protein synthesis at unassigned AGA and AGG codons. Strong expression of ICT1 is observed in cancer cells, which allows considering ICT1 as a marker of tumor cells in medical diagnostics. Its knockdown suppresses proliferation, induces cell cycle arrest and apoptosis by regulating key signal transmission pathways. Thus, ICT1 performs structural, catalytic, and regulatory functions in human cells, bringing together such processes as protein biosynthesis in mitochondria, assembly of mitochondrial ribosomes, apoptosis, and intracellular signaling.
